# Preoperative Glucagon-like peptide-1 receptor imaging reduces surgical trauma and pancreatic tissue loss in insulinoma patients: a report of three cases

**DOI:** 10.1186/s13037-015-0064-7

**Published:** 2015-06-02

**Authors:** Anna Silvia Wenning, Paul Kirchner, Kwadwo Antwi, Melpomeni Fani, Damian Wild, Emanuel Christ, Beat Gloor

**Affiliations:** Department of Visceral Surgery and Medicine, Inselspital, University Hospital, CH-3010 Berne, Switzerland; Division of Endocrinology, Diabetology and Clinical Nutrition, Inselspital, University Hospital, Berne, Switzerland; Department of Radiology, Division of Nuclear Medicine, University Hospital, Basel, Switzerland

## Abstract

**Background:**

Insulinomas are rare tumors, in the majority of cases best treated by surgical resection. Preoperative localization of insulinoma is challenging. The more precise the preoperative localization the less invasive and safer is the resection. The purpose of the study is to check the impact of a new technique to localize insulinoma on the surgical strategy.

**Findings:**

We present exact preoperative localization with Glucagon-like peptide-1 receptor (GLP-1R) imaging. This allows a more precise resection thereby reducing surgical access trauma, loss of healthy pancreatic tissue and increasing safety and quality of the surgical intervention.

**Conclusion:**

With the help of precise preoperative localization of insulinoma with GLP-1R imaging the surgeon is able to minimize the amount of resected healthy pancreatic tissue. We hypothesize that GLP-1R imaging will become a preoperative diagnostic tool to be used for many patients scheduled for open or laparoscopic insulinoma resection.

## Introduction

Precise preoperative localization of insulinoma is challenging. In 90% insulinomas are located in the pancreas, most of them with a size below 2 cm. In a recent systematic review computed tomography (CT) is described as diagnostic modality of choice and reaches a rate of correct localization of 44.4% [1]. Magnetic resonance imaging (MRI) is an accepted alternative with a rate of correct localization of 47.4%. The sensitivity showed a wide variation between 2–95.3% for CT (mean 43.9%) and 0-100% for MRI (mean 53.3). With intraoperative ultrasound insulinomas of a size of 2-3 mm can be detected [2]. The rate of correct localization was reviewed as 91.5% with a mean sensitivity of 91.2%. Therefore, open (or laparoscopic) surgical exploration combined with intraoperative ultrasound remains the preferred approach to localize insulinoma. Unfortunately, with this technique the entire pancreas needs to be surgically exposed if the preoperative CT and or MRI do not exactly show the localization of the insulinoma.

Glucagon-like peptide-1 receptor (GLP-1R) imaging using ^111^In-exendin-4 SPECT/CT has been shown to be more sensitive in detection of insulinoma than CT or MRI [3-5]. Until now, only case series have been published. In detail, in 2008 6 patients had a 100% correct preoperative localization rate. The following prospective multicenter study showed a sensitivity of 95% for the GLP-1R imaging in contrast to 47% for CT or MRI. Especially in patients without pathological findings in routine diagnostic GLP-1R imaging was helpful to localize insulinoma. However it is important to know that many malign insulinomas lack GLP-1R and will not be detectable by GLP-1R targeted imaging.

We hypothesize that exact preoperative localization with GLP-1R imaging allows for a more precise resection thereby reducing surgical access trauma, loss of healthy pancreatic tissue and increasing safety and quality of the surgical intervention.

## Methods

We present three consecutive cases of insulinomas which are not included in previously published series and focus on surgical tactics and safety. Prior to surgery, all patients underwent GLP-1R imaging using ^111^In-DOTA-exendin-4 SPECT/CT in addition to CT or MRI. Synthesis and labelling of ^111^In-DOTA-exendin-4 was published elsewhere [4]. SPECT/CT of the abdomen was performed at 4 and 72 hours after i.v. injection of ^111^In-DOTA-exendin-4. Surgery was done by one single surgical team (ASW and BG).

## Results

**Figure 1:**
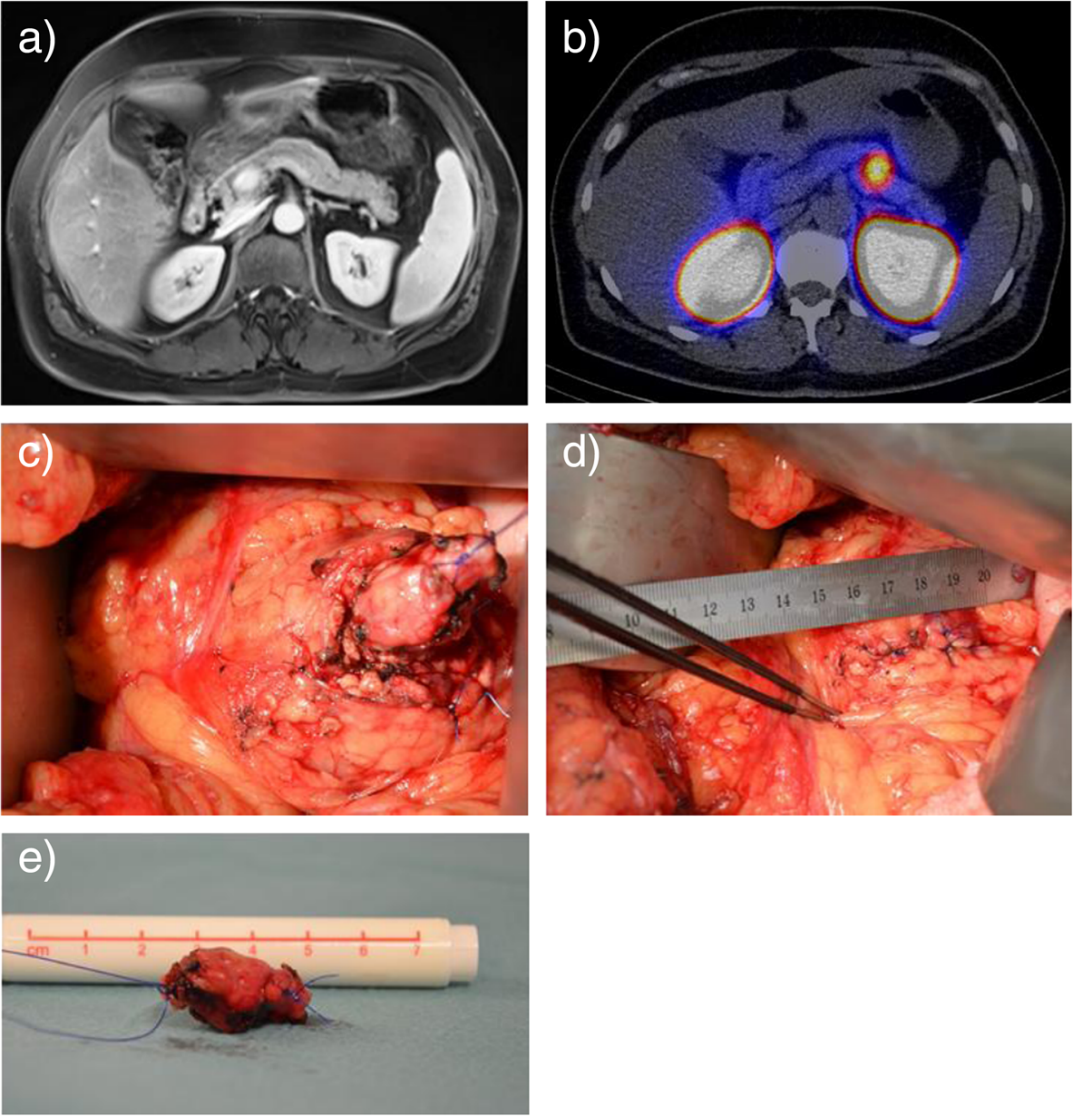
**a)** and **b)** MRI without pathological finding in the pancreatic corpus whereas ^111^In-DOTA-exndin-4 SPECT/CT detects the insulinoma. **c)** Enucleation was performed and **d)** the pancreatic capsule was closed by direct suture. **e)** The insulinoma (14 mm) was surrounded by a minimal mass of healthy tissue.

In the first patient, MRI showed a small hypervascular lesion in the uncinate process and a normal pancreatic corpus. In contrast GLP-1R imaging using ^111^In-DOTA-exendin-4 SPECT/CT detected an insulinoma in the pancreatic corpus (Figure 1a and b). Surgical exploration was performed with an open access. Intraoperative inspection and palpation confirmed the central tumor that was enucleated (Figure 1c,d and e). Histology showed a 14 mm insulinoma. No further lesions were found in the uncinate process.

**Figure 2:**
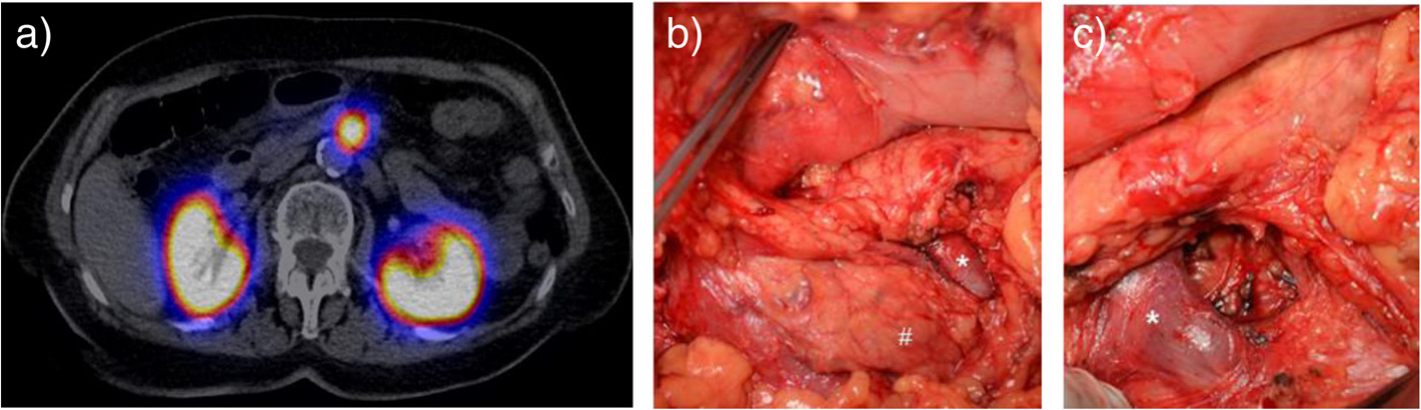
**a)** ^111^In-DOTA-exendin-4 SPECT/CT localized the insulinoma between superior mesenteric artery and vein. **b)** Open exploration confirmed this localization behind the superior mesenteric vein and **c)** the tumor was enucleated. (*: superior mesenteric vein, #: uncinate process).

MRI of the second patient showed no pathologies of the pancreas. Only GLP-1R imaging detected a lesion dorsal of the uncinate process in projection of the interspace between superior mesenteric vein and artery (Figure 2a). The open approach focused on preparation of the uncinate process and the superior mesenteric vein. Enucleation of a small tumor was performed without complications (Figure 2b and c) and confirmed histologically.

**Figure 3:**
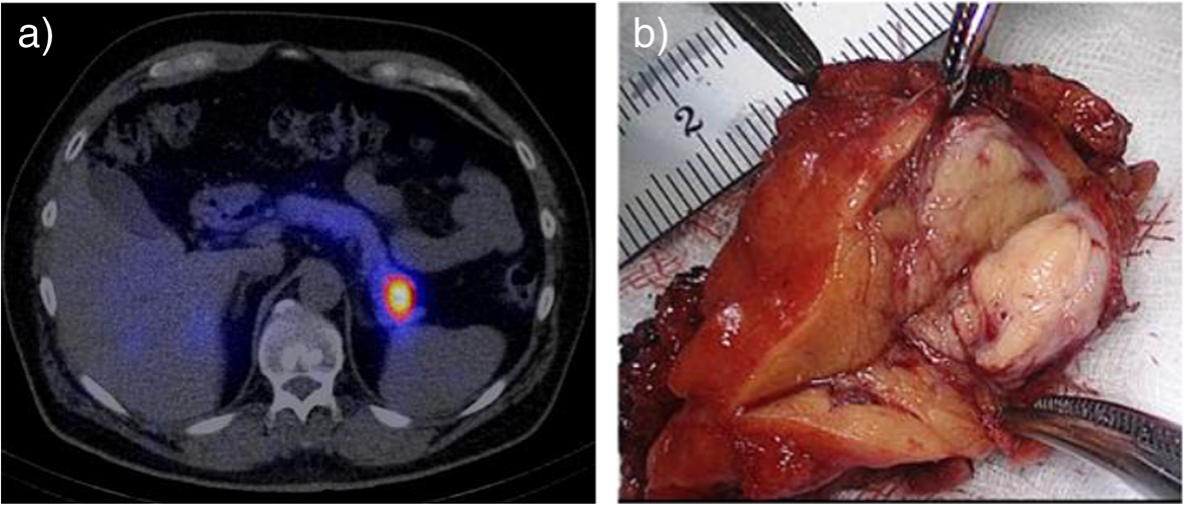
**a)** A large insulinoma of the pancreatic tail was detected with ^111^In-DOTA-exendin-4 SPECT/CT and **b)** resected laparoscopically (pancreatic tail resection).

The third patient underwent CT and MRI. Both modalities did not detect the insulinoma of 1.6 cm in the pancreatic tail, that became visible in GLP-1R imaging (Figure 3a). Pancreatic tail resection was performed laparoscopically. The tumor was sent to frozen section and verified (Figure 3b) allowing for omission of exposure of the body and head of the pancreas.

None of the three patients developed postoperative complications (especially no pancreatic fistula and no diabetes mellitus). No persisting hypoglycemia occurred. A summary of results of the diagnostic test and intraoperative findings is given in Table 1.

**Table 1:** **Preoperative imaging to localize insulinoma, intraoperative finding and surgical approach in three patients**

**Imaging/surgical procedure**	**Patient 1 (f)**	**Patient 2 (f)**	**Patient 3 (m)**
CT scan	-	-	Normal
MRI	Hypervascular lesion in uncinate process	Normal	Normal
^111^In-DOTA-exendin-4 SPECT/CT	Pancreatic corpus	Dorsal of uncinate process	Pancreatic tail
Intra-operative	Pancreatic corpus	Dorsal of uncinate process	Pancreatic tail
Access	Open	Open	Laparoscopic
Technique	Enucleation	Enucleation	Pancreatic tail resection

## Discussion

In all three patients GLP-1R imaging was crucial for planning the resection. Operation was performed pointing straight to the localization of interest. The access was kept as minimal as possible und healthy pancreatic tissue was preserved.

In the first patient preoperative imaging did not show the neuroendocrine tumor but falsely found a lesion in the uncinate process. With the help of the GLP-1R imaging we were able to keep surgical exploration of the uncinate process at a minimum.

In the third patient GLP-1R imaging led to laparoscopic resection. Laparoscopic sonography is an established method, but digital palpation cannot be used. Therefore only a reliable preoperative localization of insulinoma allows a safe and fast laparoscopic approach.

Based on these three cases and the previously published data we recommend preoperative GLP-1R imaging in insulinoma patients. Especially in ectopic or small lesions below a size of 1 cm and in MEN1 patients who sometimes suffer from multiple insulinomas or if preoperative imaging does not at all or not correctly show the lesion the benefit for our patients is high. Another advantage is the possibility to use the gamma-probe to detect an insulinoma intraoperatively, as long as the resection is planned less than 14 days after the scan [4]. With these tools the risk for an unsuccessful operation is reduced to a minimum.

In our experience the preoperative GLP-1R imaging leads to focused access to the insulinoma, preservation of more normal pancreatic tissue and thus to a higher precision and safety of the procedure.

## Conclusion

In our view preoperative GLP-1R imaging is important for planning surgical treatment in insulinoma. For the future it may allow to perform laparoscopic resections more frequently.
